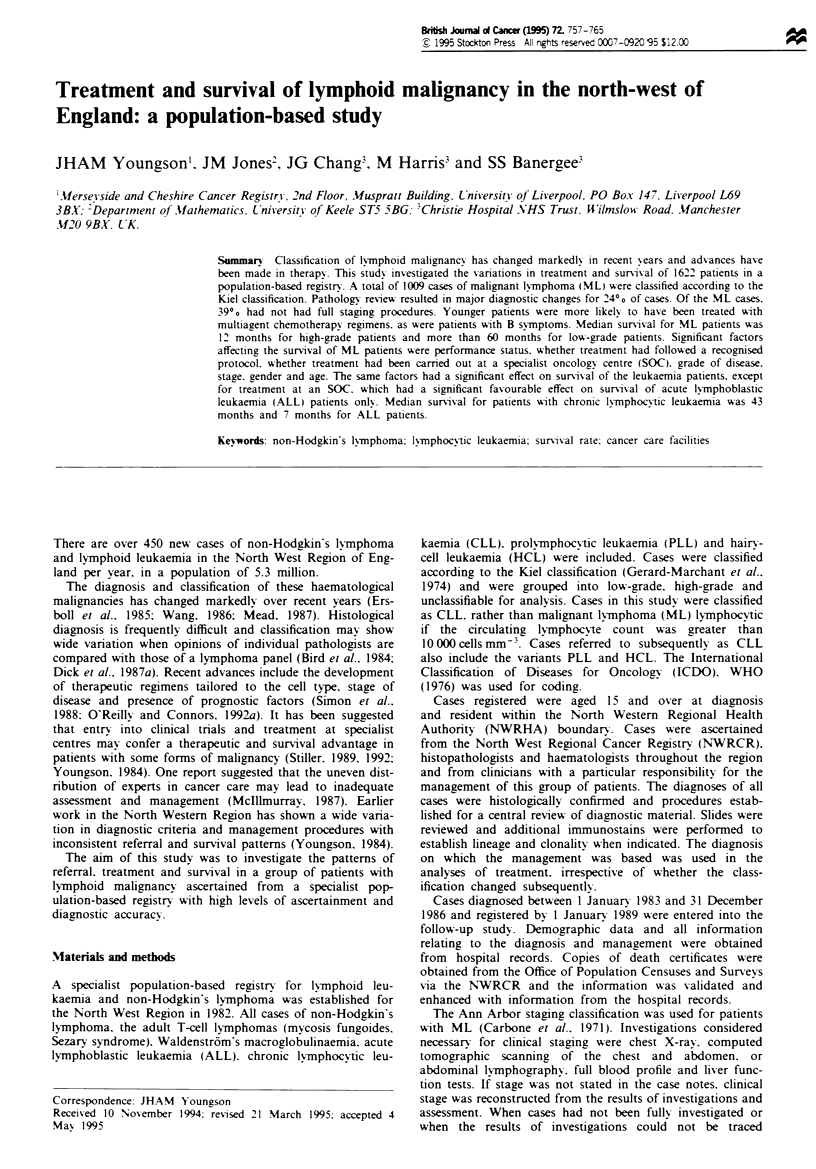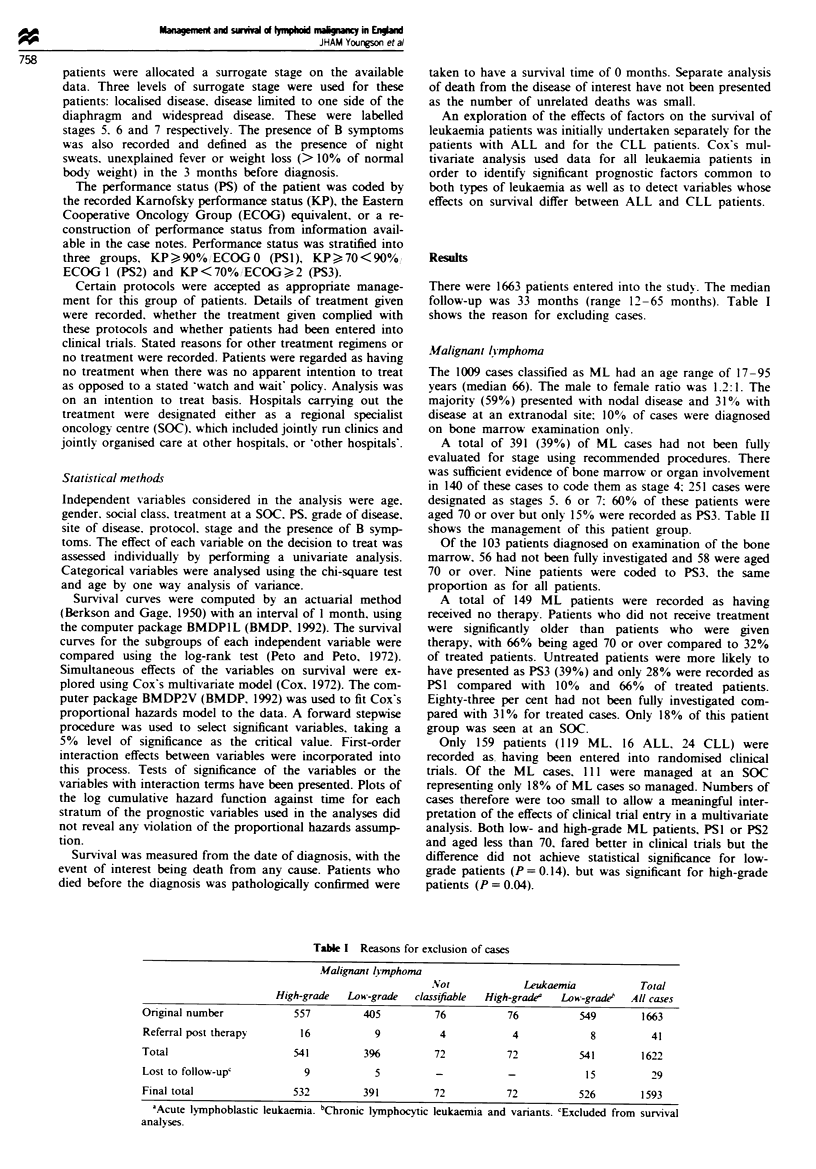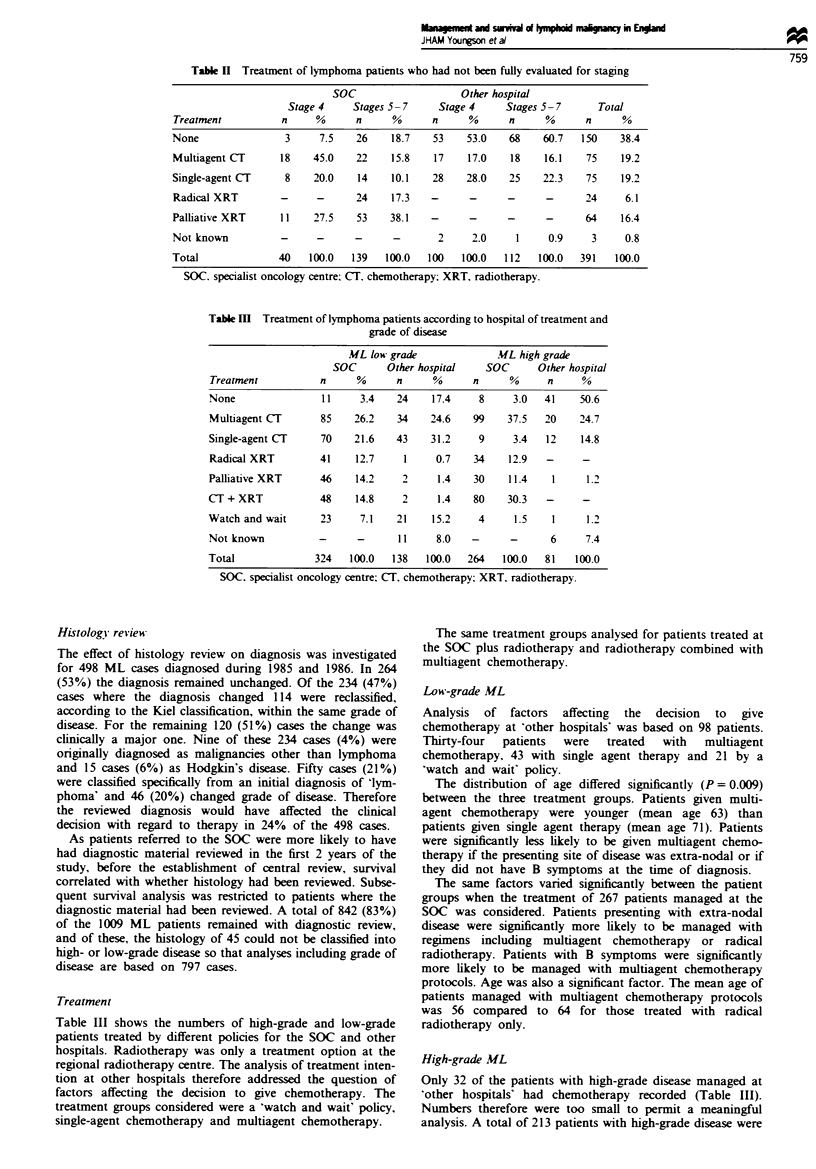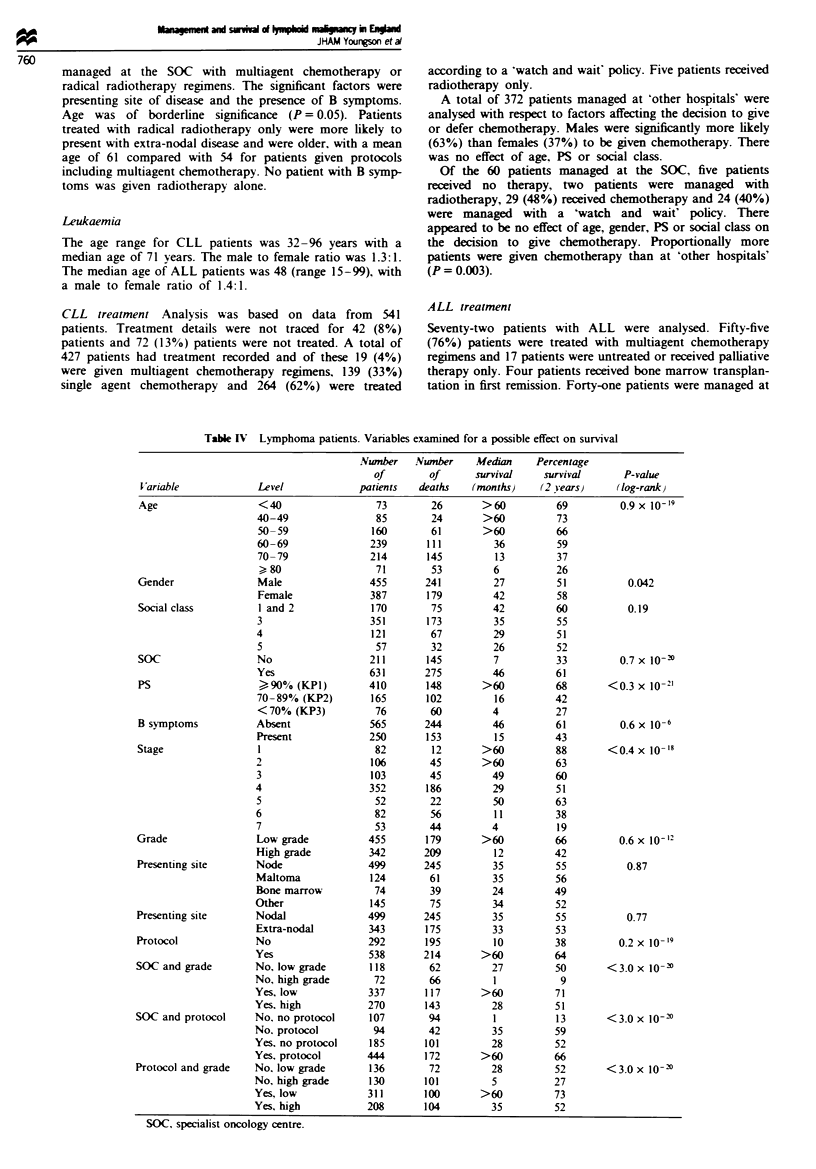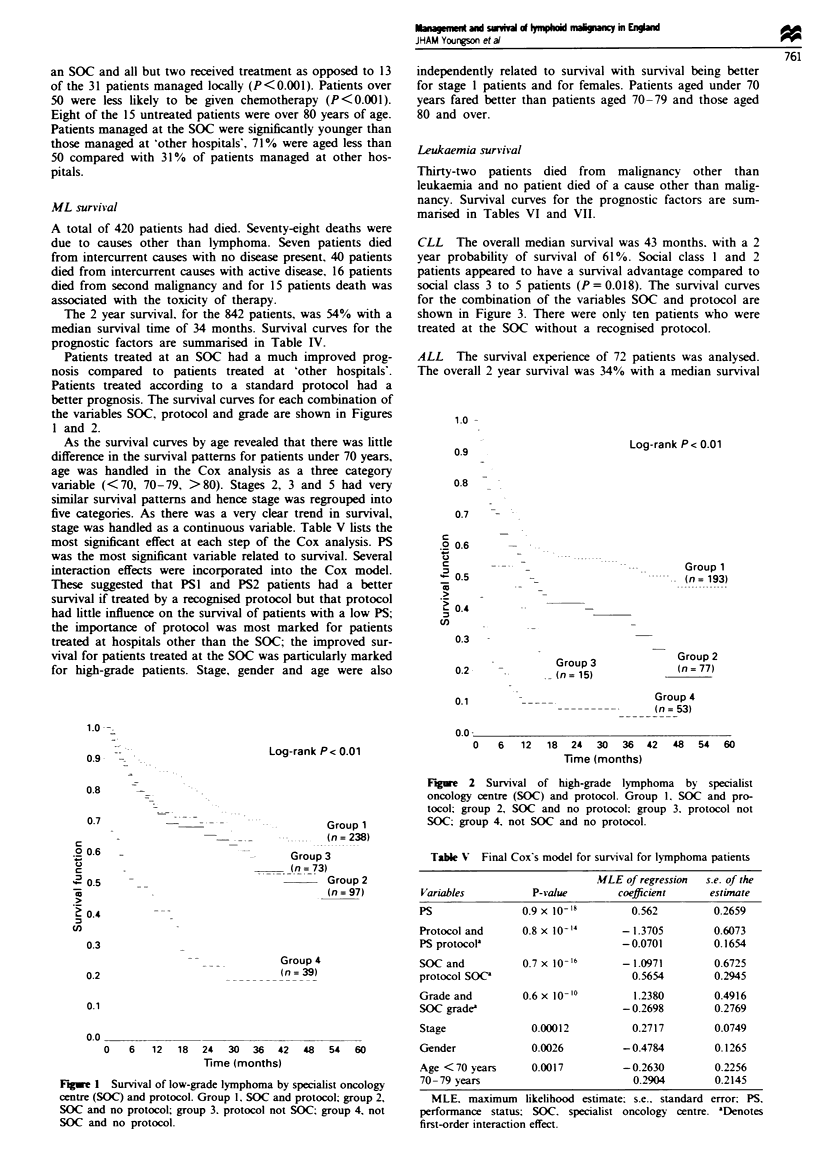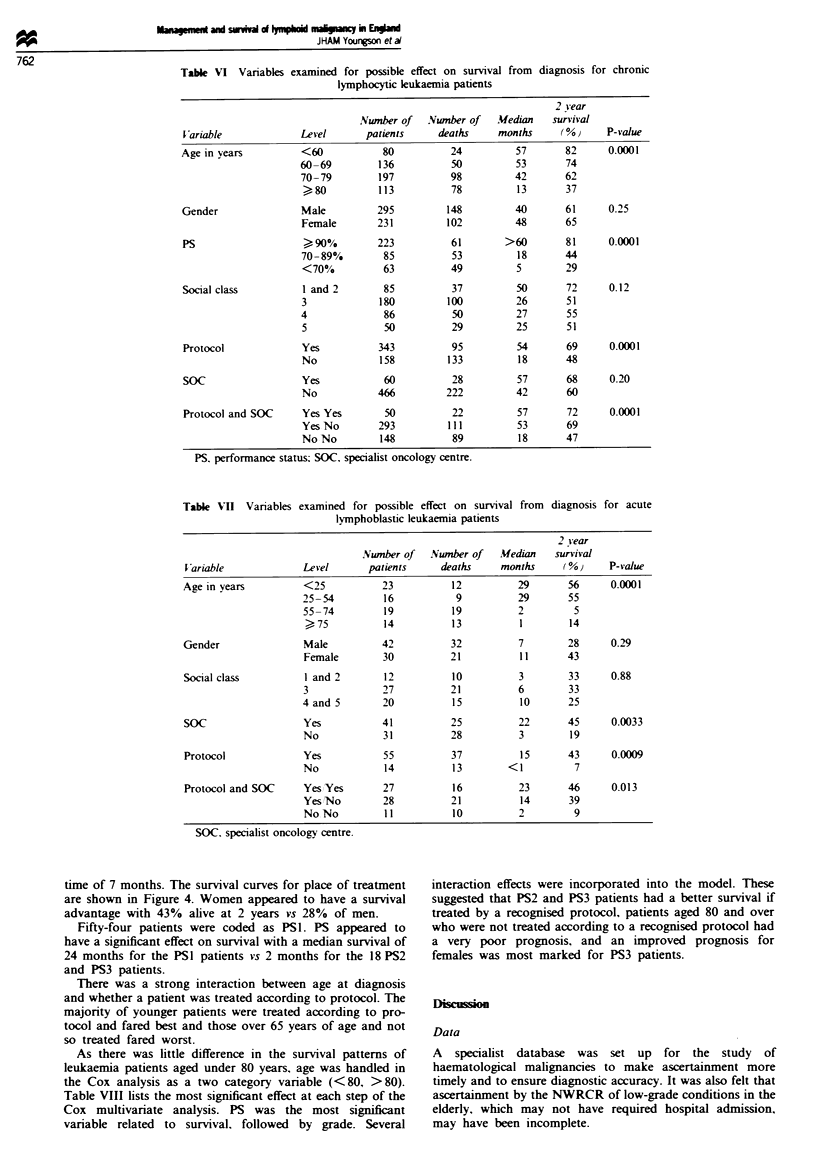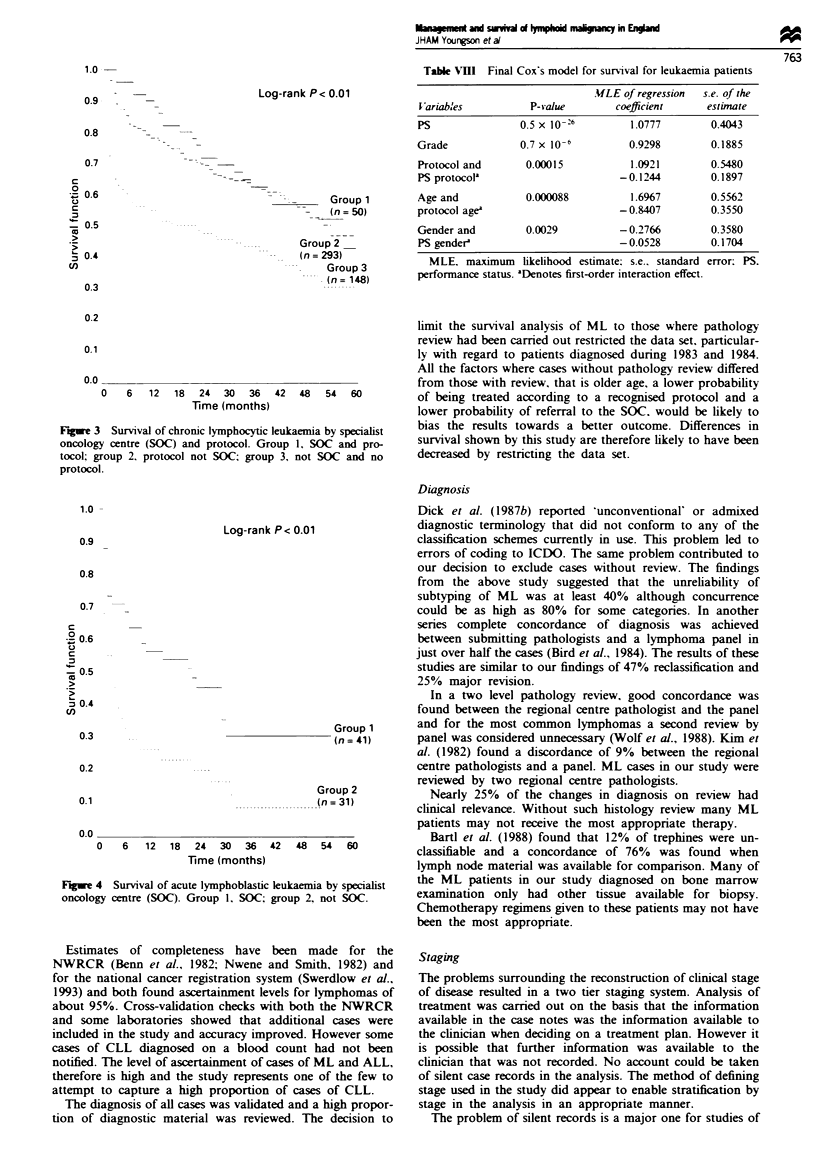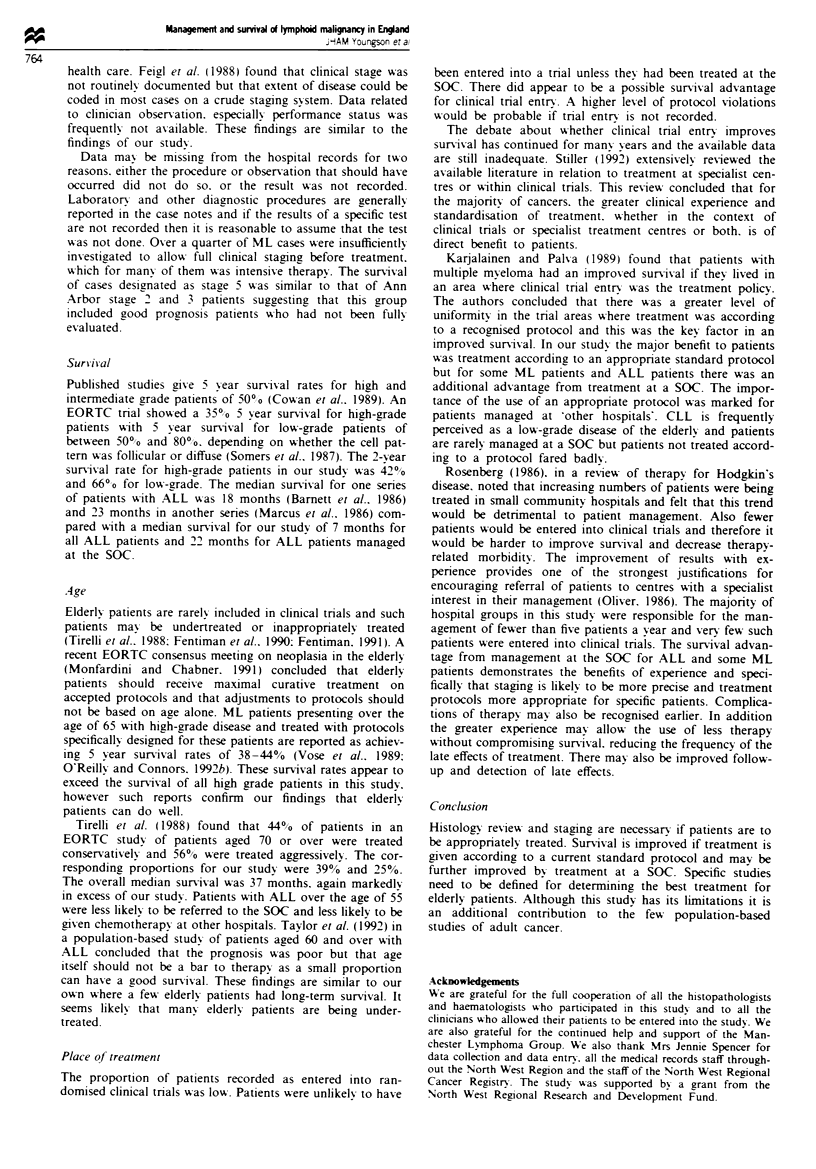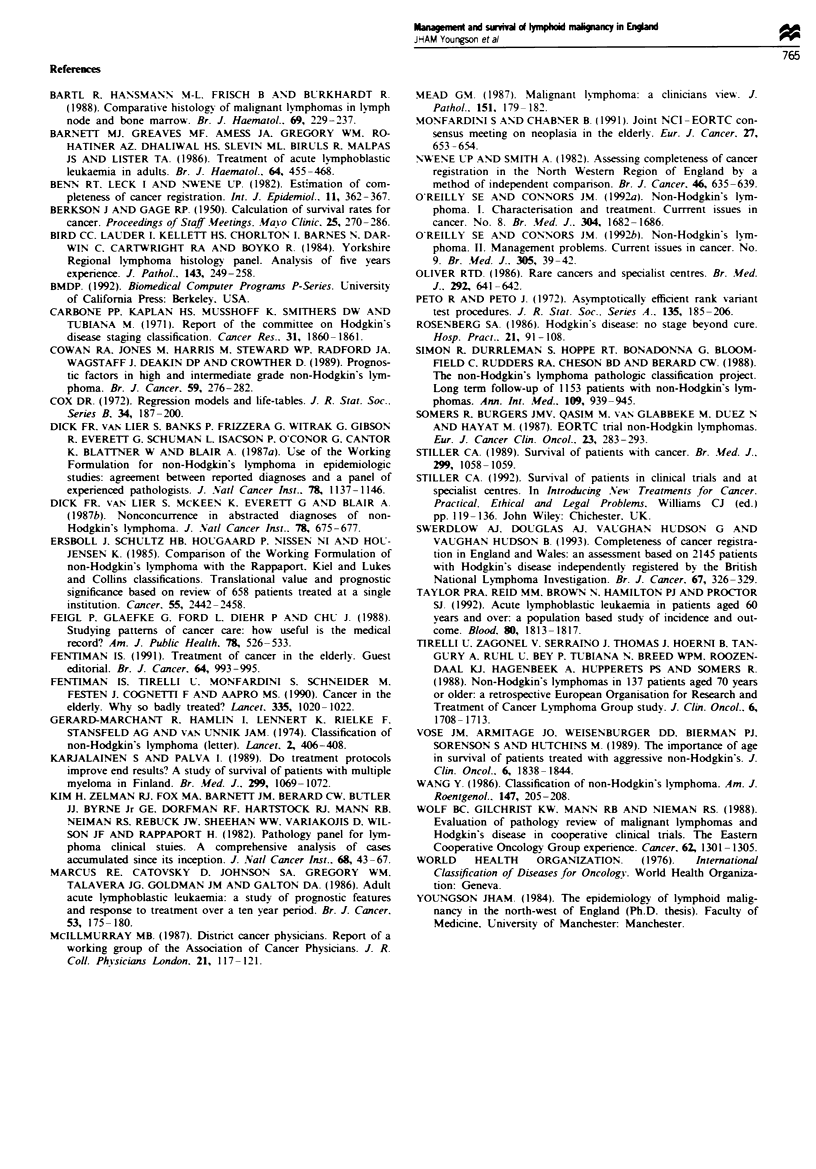# Treatment and survival of lymphoid malignancy in the north-west of England: a population-based study.

**DOI:** 10.1038/bjc.1995.406

**Published:** 1995-09

**Authors:** J. H. Youngson, J. M. Jones, J. G. Chang, M. Harris, S. S. Banergee

**Affiliations:** Merseyside and Cheshire Cancer Registry, University of Liverpool, UK.

## Abstract

Classification of lymphoid malignancy has changed markedly in recent years and advances have been made in therapy. This study investigated the variations in treatment and survival of 1622 patients in a population-based registry. A total of 1009 cases of malignant lymphoma (ML) were classified according to the Kiel classification. Pathology review resulted in major diagnostic changes for 24% of cases. Of the ML cases, 39% had not had full staging procedures. Younger patients were more likely to have been treated with multiagent chemotherapy regimens, as were patients with B symptoms. Median survival for ML patients was 12 months for high-grade patients and more than 60 months for low-grade patients. Significant factors affecting the survival of ML patients were performance status, whether treatment had followed a recognised protocol, whether treatment had been carried out at a specialist oncology centre (SOC), grade of disease, stage, gender and age. The same factors had a significant effect on survival of the leukaemia patients, except for treatment at an SOC, which had a significant favourable effect on survival of acute lymphoblastic leukaemia (ALL) patients only. Median survival for patients with chronic lymphocytic leukaemia was 43 months and 7 months for ALL patients.


British Journal of Cancer (1995) 72, 757-765

© 1995 Stockton Press All rights reserved 0007-0920/95 $12.00

### Treatment and survival of lymphoid malignancy in the north-west of England: a population-based study

JHAM Youngson[1], JM Jones[2], JG Chang[3], M Harris[3] and SS Banergee[3]

[1]*Merseyside and Cheshire Cancer Registry, 2nd Floor, Muspratt Building, University of Liverpool, PO Box 147, Liverpool L69 3BX;* [2]*Department of Mathematics, University of Keele ST5 5BG;* [3]*Christie Hospital NHS Trust, Wilmslow Road, Manchester M20 9BX, UK.*

**Summary** Classification of lymphoid malignancy has changed markedly in recent years and advances have been made in therapy. This study investigated the variations in treatment and survival of 1622 patients in a population-based registry. A total of 1009 cases of malignant lymphoma (ML) were classified according to the Kiel classification. Pathology review resulted in major diagnostic changes for 24% of cases. Of the ML cases, 39% had not had full staging procedures. Younger patients were more likely to have been treated with multiagent chemotherapy regimens, as were patients with B symptoms. Median survival for ML patients was 12 months for high-grade patients and more than 60 months for low-grade patients. Significant factors affecting the survival of ML patients were performance status, whether treatment had followed a recognised protocol, whether treatment had been carried out at a specialist oncology centre (SOC), grade of disease, stage, gender and age. The same factors had a significant effect on survival of the leukaemia patients, except for treatment at an SOC, which had a significant favourable effect on survival of acute lymphoblastic leukaemia (ALL) patients only. Median survival for patients with chronic lymphocytic leukaemia was 43 months and 7 months for ALL patients.

**Keywords**: non-Hodgkin's lymphoma; lymphocytic leukaemia; survival rate; cancer care facilities

There are over 450 new cases of non-Hodgkin's lymphoma and lymphoid leukaemia in the North West Region of England per year, in a population of 5.3 million.

The diagnosis and classification of these haematological malignancies has changed markedly over recent years (Ersboll *et al.*, 1985; Wang, 1986; Mead, 1987). Histological diagnosis is frequently difficult and classification may show wide variation when opinions of individual pathologists are compared with those of a lymphoma panel (Bird *et al.*, 1984; Dick *et al.*, 1987*a*). Recent advances include the development of therapeutic regimens tailored to the cell type, stage of disease and presence of prognostic factors (Simon *et al.*, 1988; O'Reilly and Connors, 1992*a*). It has been suggested that entry into clinical trials and treatment at specialist centres may confer a therapeutic and survival advantage in patients with some forms of malignancy (Stiller, 1989, 1992; Youngson, 1984). One report suggested that the uneven distribution of experts in cancer care may lead to inadequate assessment and management (McIllmurray, 1987). Earlier work in the North Western Region has shown a wide variation in diagnostic criteria and management procedures with inconsistent referral and survival patterns (Youngson, 1984).

The aim of this study was to investigate the patterns of referral, treatment and survival in a group of patients with lymphoid malignancy ascertained from a specialist population-based registry with high levels of ascertainment and diagnostic accuracy.

### Materials and methods

A specialist population-based registry for lymphoid leukaemia and non-Hodgkin's lymphoma was established for the North West Region in 1982. All cases of non-Hodgkin's lymphoma, the adult T-cell lymphomas (mycosis fungoides, Sezary syndrome), Waldenström's macroglobulinaemia, acute lymphoblastic leukaemia (ALL), chronic lymphocytic leukaemia (CLL), prolymphocytic leukaemia (PLL) and hairy-cell leukaemia (HCL) were included. Cases were classified according to the Kiel classification (Gerard-Marchant *et al.*, 1974) and were grouped into low-grade, high-grade and unclassifiable for analysis. Cases in this study were classified as CLL, rather than malignant lymphoma (ML) lymphocytic if the circulating lymphocyte count was greater than 10 000 cells $mm^{-3}$. Cases referred to subsequently as CLL also include the variants PLL and HCL. The International Classification of Diseases for Oncology (ICDO), WHO (1976) was used for coding.

Cases registered were aged 15 and over at diagnosis and resident within the North Western Regional Health Authority (NWRHA) boundary. Cases were ascertained from the North West Regional Cancer Registry (NWRCR), histopathologists and haematologists throughout the region and from clinicians with a particular responsibility for the management of this group of patients. The diagnoses of all cases were histologically confirmed and procedures established for a central review of diagnostic material. Slides were reviewed and additional immunostains were performed to establish lineage and clonality when indicated. The diagnosis on which the management was based was used in the analyses of treatment, irrespective of whether the classification changed subsequently.

Cases diagnosed between 1 January 1983 and 31 December 1986 and registered by 1 January 1989 were entered into the follow-up study. Demographic data and all information relating to the diagnosis and management were obtained from hospital records. Copies of death certificates were obtained from the Office of Population Censuses and Surveys via the NWRCR and the information was validated and enhanced with information from the hospital records.

The Ann Arbor staging classification was used for patients with ML (Carbone *et al.*, 1971). Investigations considered necessary for clinical staging were chest X-ray, computed tomographic scanning of the chest and abdomen, or abdominal lymphography, full blood profile and liver function tests. If stage was not stated in the case notes, clinical stage was reconstructed from the results of investigations and assessment. When cases had not been fully investigated or when the results of investigations could not be traced

Correspondence: JHAM Youngson
Received 10 November 1994; revised 21 March 1995; accepted 4 May 1995

patients were allocated a surrogate stage on the available data. Three levels of surrogate stage were used for these patients: localised disease, disease limited to one side of the diaphragm and widespread disease. These were labelled stages 5, 6 and 7 respectively. The presence of B symptoms was also recorded and defined as the presence of night sweats, unexplained fever or weight loss (>10% of normal body weight) in the 3 months before diagnosis.

The performance status (PS) of the patient was coded by the recorded Karnofsky performance status (KP), the Eastern Cooperative Oncology Group (ECOG) equivalent, or a reconstruction of performance status from information available in the case notes. Performance status was stratified into three groups, KP≥90%/ECOG 0 (PS1), KP≥70<90%/ECOG 1 (PS2) and KP<70%/ECOG≥2 (PS3).

Certain protocols were accepted as appropriate management for this group of patients. Details of treatment given were recorded, whether the treatment given complied with these protocols and whether patients had been entered into clinical trials. Stated reasons for other treatment regimens or no treatment were recorded. Patients were regarded as having no treatment when there was no apparent intention to treat as opposed to a stated 'watch and wait' policy. Analysis was on an intention to treat basis. Hospitals carrying out the treatment were designated either as a regional specialist oncology centre (SOC), which included jointly run clinics and jointly organised care at other hospitals, or 'other hospitals'.

*Statistical methods*

Independent variables considered in the analysis were age, gender, social class, treatment at a SOC, PS, grade of disease, site of disease, protocol, stage and the presence of B symptoms. The effect of each variable on the decision to treat was assessed individually by performing a univariate analysis. Categorical variables were analysed using the chi-square test and age by one way analysis of variance.

Survival curves were computed by an actuarial method (Berkson and Gage, 1950) with an interval of 1 month, using the computer package BMDP1L (BMDP, 1992). The survival curves for the subgroups of each independent variable were compared using the log-rank test (Peto and Peto, 1972). Simultaneous effects of the variables on survival were explored using Cox's multivariate model (Cox, 1972). The computer package BMDP2V (BMDP, 1992) was used to fit Cox's proportional hazards model to the data. A forward stepwise procedure was used to select significant variables, taking a 5% level of significance as the critical value. First-order interaction effects between variables were incorporated into this process. Tests of significance of the variables or the variables with interaction terms have been presented. Plots of the log cumulative hazard function against time for each stratum of the prognostic variables used in the analyses did not reveal any violation of the proportional hazards assumption.

Survival was measured from the date of diagnosis, with the event of interest being death from any cause. Patients who died before the diagnosis was pathologically confirmed were taken to have a survival time of 0 months. Separate analysis of death from the disease of interest have not been presented as the number of unrelated deaths was small.

An exploration of the effects of factors on the survival of leukaemia patients was initially undertaken separately for the patients with ALL and for the CLL patients. Cox's multivariate analysis used data for all leukaemia patients in order to identify significant prognostic factors common to both types of leukaemia as well as to detect variables whose effects on survival differ between ALL and CLL patients.

### Results

There were 1663 patients entered into the study. The median follow-up was 33 months (range 12–65 months). Table I shows the reason for excluding cases.

*Malignant lymphoma*

The 1009 cases classified as ML had an age range of 17–95 years (median 66). The male to female ratio was 1.2:1. The majority (59%) presented with nodal disease and 31% with disease at an extranodal site; 10% of cases were diagnosed on bone marrow examination only.

A total of 391 (39%) of ML cases had not been fully evaluated for stage using recommended procedures. There was sufficient evidence of bone marrow or organ involvement in 140 of these cases to code them as stage 4; 251 cases were designated as stages 5, 6 or 7; 60% of these patients were aged 70 or over but only 15% were recorded as PS3. Table II shows the management of this patient group.

Of the 103 patients diagnosed on examination of the bone marrow, 56 had not been fully investigated and 58 were aged 70 or over. Nine patients were coded to PS3, the same proportion as for all patients.

A total of 149 ML patients were recorded as having received no therapy. Patients who did not receive treatment were significantly older than patients who were given therapy, with 66% being aged 70 or over compared to 32% of treated patients. Untreated patients were more likely to have presented as PS3 (39%) and only 28% were recorded as PS1 compared with 10% and 66% of treated patients. Eighty-three per cent had not been fully investigated compared with 31% for treated cases. Only 18% of this patient group was seen at an SOC.

Only 159 patients (119 ML, 16 ALL, 24 CLL) were recorded as having been entered into randomised clinical trials. Of the ML cases, 111 were managed at an SOC representing only 18% of ML cases so managed. Numbers of cases therefore were too small to allow a meaningful interpretation of the effects of clinical trial entry in a multivariate analysis. Both low- and high-grade ML patients, PS1 or PS2 and aged less than 70, fared better in clinical trials but the difference did not achieve statistical significance for low-grade patients ($P = 0.14$), but was significant for high-grade patients ($P = 0.04$).

**Table I** Reasons for exclusion of cases

| | *Malignant lymphoma* | | | *Leukaemia* | | |
|---|---|---|---|---|---|---|
| | *High-grade* | *Low-grade* | *Not classifiable* | *High-grade*[a] | *Low-grade*[b] | *Total All cases* |
| Original number | 557 | 405 | 76 | 76 | 549 | 1663 |
| Referral post therapy | 16 | 9 | 4 | 4 | 8 | 41 |
| Total | 541 | 396 | 72 | 72 | 541 | 1622 |
| Lost to follow-up[c] | 9 | 5 | – | – | 15 | 29 |
| Final total | 532 | 391 | 72 | 72 | 526 | 1593 |

[a]Acute lymphoblastic leukaemia. [b]Chronic lymphocytic leukaemia and variants. [c]Excluded from survival analyses.

**Table II** Treatment of lymphoma patients who had not been fully evaluated for staging

| | SOC | | | | Other hospital | | | | | |
|---|---|---|---|---|---|---|---|---|---|---|
| | Stage 4 | | Stages 5–7 | | Stage 4 | | Stages 5–7 | | Total | |
| Treatment | n | % | n | % | n | % | n | % | n | % |
| None | 3 | 7.5 | 26 | 18.7 | 53 | 53.0 | 68 | 60.7 | 150 | 38.4 |
| Multiagent CT | 18 | 45.0 | 22 | 15.8 | 17 | 17.0 | 18 | 16.1 | 75 | 19.2 |
| Single-agent CT | 8 | 20.0 | 14 | 10.1 | 28 | 28.0 | 25 | 22.3 | 75 | 19.2 |
| Radical XRT | – | – | 24 | 17.3 | – | – | – | – | 24 | 6.1 |
| Palliative XRT | 11 | 27.5 | 53 | 38.1 | – | – | – | – | 64 | 16.4 |
| Not known | – | – | – | – | 2 | 2.0 | 1 | 0.9 | 3 | 0.8 |
| Total | 40 | 100.0 | 139 | 100.0 | 100 | 100.0 | 112 | 100.0 | 391 | 100.0 |

SOC, specialist oncology centre; CT, chemotherapy; XRT, radiotherapy.

**Table III** Treatment of lymphoma patients according to hospital of treatment and grade of disease

| | ML low grade | | | | ML high grade | | | |
|---|---|---|---|---|---|---|---|---|
| | SOC | | Other hospital | | SOC | | Other hospital | |
| Treatment | n | % | n | % | n | % | n | % |
| None | 11 | 3.4 | 24 | 17.4 | 8 | 3.0 | 41 | 50.6 |
| Multiagent CT | 85 | 26.2 | 34 | 24.6 | 99 | 37.5 | 20 | 24.7 |
| Single-agent CT | 70 | 21.6 | 43 | 31.2 | 9 | 3.4 | 12 | 14.8 |
| Radical XRT | 41 | 12.7 | 1 | 0.7 | 34 | 12.9 | – | – |
| Palliative XRT | 46 | 14.2 | 2 | 1.4 | 30 | 11.4 | 1 | 1.2 |
| CT + XRT | 48 | 14.8 | 2 | 1.4 | 80 | 30.3 | – | – |
| Watch and wait | 23 | 7.1 | 21 | 15.2 | 4 | 1.5 | 1 | 1.2 |
| Not known | – | – | 11 | 8.0 | – | – | 6 | 7.4 |
| Total | 324 | 100.0 | 138 | 100.0 | 264 | 100.0 | 81 | 100.0 |

SOC, specialist oncology centre; CT, chemotherapy; XRT, radiotherapy.

### *Histology review*

The effect of histology review on diagnosis was investigated for 498 ML cases diagnosed during 1985 and 1986. In 264 (53%) the diagnosis remained unchanged. Of the 234 (47%) cases where the diagnosis changed 114 were reclassified, according to the Kiel classification, within the same grade of disease. For the remaining 120 (51%) cases the change was clinically a major one. Nine of these 234 cases (4%) were originally diagnosed as malignancies other than lymphoma and 15 cases (6%) as Hodgkin's disease. Fifty cases (21%) were classified specifically from an initial diagnosis of 'lymphoma' and 46 (20%) changed grade of disease. Therefore the reviewed diagnosis would have affected the clinical decision with regard to therapy in 24% of the 498 cases.

As patients referred to the SOC were more likely to have had diagnostic material reviewed in the first 2 years of the study, before the establishment of central review, survival correlated with whether histology had been reviewed. Subsequent survival analysis was restricted to patients where the diagnostic material had been reviewed. A total of 842 (83%) of the 1009 ML patients remained with diagnostic review, and of these, the histology of 45 could not be classified into high- or low-grade disease so that analyses including grade of disease are based on 797 cases.

### *Treatment*

Table III shows the numbers of high-grade and low-grade patients treated by different policies for the SOC and other hospitals. Radiotherapy was only a treatment option at the regional radiotherapy centre. The analysis of treatment intention at other hospitals therefore addressed the question of factors affecting the decision to give chemotherapy. The treatment groups considered were a 'watch and wait' policy, single-agent chemotherapy and multiagent chemotherapy.

The same treatment groups analysed for patients treated at the SOC plus radiotherapy and radiotherapy combined with multiagent chemotherapy.

### *Low-grade ML*

Analysis of factors affecting the decision to give chemotherapy at 'other hospitals' was based on 98 patients. Thirty-four patients were treated with multiagent chemotherapy, 43 with single agent therapy and 21 by a 'watch and wait' policy.

The distribution of age differed significantly ($P = 0.009$) between the three treatment groups. Patients given multiagent chemotherapy were younger (mean age 63) than patients given single agent therapy (mean age 71). Patients were significantly less likely to be given multiagent chemotherapy if the presenting site of disease was extra-nodal or if they did not have B symptoms at the time of diagnosis.

The same factors varied significantly between the patient groups when the treatment of 267 patients managed at the SOC was considered. Patients presenting with extra-nodal disease were significantly more likely to be managed with regimens including multiagent chemotherapy or radical radiotherapy. Patients with B symptoms were significantly more likely to be managed with multiagent chemotherapy protocols. Age was also a significant factor. The mean age of patients managed with multiagent chemotherapy protocols was 56 compared to 64 for those treated with radical radiotherapy only.

### *High-grade ML*

Only 32 of the patients with high-grade disease managed at 'other hospitals' had chemotherapy recorded (Table III). Numbers therefore were too small to permit a meaningful analysis. A total of 213 patients with high-grade disease were managed at the SOC with multiagent chemotherapy or radical radiotherapy regimens. The significant factors were presenting site of disease and the presence of B symptoms. Age was of borderline significance ($P = 0.05$). Patients treated with radical radiotherapy only were more likely to present with extra-nodal disease and were older, with a mean age of 61 compared with 54 for patients given protocols including multiagent chemotherapy. No patient with B symptoms was given radiotherapy alone.

### *Leukaemia*

The age range for CLL patients was 32–96 years with a median age of 71 years. The male to female ratio was 1.3:1. The median age of ALL patients was 48 (range 15–99), with a male to female ratio of 1.4:1.

*CLL treatment* Analysis was based on data from 541 patients. Treatment details were not traced for 42 (8%) patients and 72 (13%) patients were not treated. A total of 427 patients had treatment recorded and of these 19 (4%) were given multiagent chemotherapy regimens, 139 (33%) single agent chemotherapy and 264 (62%) were treated according to a 'watch and wait' policy. Five patients received radiotherapy only.

A total of 372 patients managed at 'other hospitals' were analysed with respect to factors affecting the decision to give or defer chemotherapy. Males were significantly more likely (63%) than females (37%) to be given chemotherapy. There was no effect of age, PS or social class.

Of the 60 patients managed at the SOC, five patients received no therapy, two patients were managed with radiotherapy, 29 (48%) received chemotherapy and 24 (40%) were managed with a 'watch and wait' policy. There appeared to be no effect of age, gender, PS or social class on the decision to give chemotherapy. Proportionally more patients were given chemotherapy than at 'other hospitals' ($P = 0.003$).

### *ALL treatment*

Seventy-two patients with ALL were analysed. Fifty-five (76%) patients were treated with multiagent chemotherapy regimens and 17 patients were untreated or received palliative therapy only. Four patients received bone marrow transplantation in first remission. Forty-one patients were managed at

**Table IV** Lymphoma patients. Variables examined for a possible effect on survival

| *Variable* | *Level* | *Number of patients* | *Number of deaths* | *Median survival (months)* | *Percentage survival (2 years)* | *P-value (log-rank)* |
|---|---|---|---|---|---|---|
| Age | <40 | 73 | 26 | >60 | 69 | $0.9 \times 10^{-19}$ |
| | 40–49 | 85 | 24 | >60 | 73 | |
| | 50–59 | 160 | 61 | >60 | 66 | |
| | 60–69 | 239 | 111 | 36 | 59 | |
| | 70–79 | 214 | 145 | 13 | 37 | |
| | ⩾80 | 71 | 53 | 6 | 26 | |
| Gender | Male | 455 | 241 | 27 | 51 | 0.042 |
| | Female | 387 | 179 | 42 | 58 | |
| Social class | 1 and 2 | 170 | 75 | 42 | 60 | 0.19 |
| | 3 | 351 | 173 | 35 | 55 | |
| | 4 | 121 | 67 | 29 | 51 | |
| | 5 | 57 | 32 | 26 | 52 | |
| SOC | No | 211 | 145 | 7 | 33 | $0.7 \times 10^{-20}$ |
| | Yes | 631 | 275 | 46 | 61 | |
| PS | ⩾90% (KP1) | 410 | 148 | >60 | 68 | $<0.3 \times 10^{-21}$ |
| | 70–89% (KP2) | 165 | 102 | 16 | 42 | |
| | <70% (KP3) | 76 | 60 | 4 | 27 | |
| B symptoms | Absent | 565 | 244 | 46 | 61 | $0.6 \times 10^{-6}$ |
| | Present | 250 | 153 | 15 | 43 | |
| Stage | 1 | 82 | 12 | >60 | 88 | $<0.4 \times 10^{-18}$ |
| | 2 | 106 | 45 | >60 | 63 | |
| | 3 | 103 | 45 | 49 | 60 | |
| | 4 | 352 | 186 | 29 | 51 | |
| | 5 | 52 | 22 | 50 | 63 | |
| | 6 | 82 | 56 | 11 | 38 | |
| | 7 | 53 | 44 | 4 | 19 | |
| Grade | Low grade | 455 | 179 | >60 | 66 | $0.6 \times 10^{-12}$ |
| | High grade | 342 | 209 | 12 | 42 | |
| Presenting site | Node | 499 | 245 | 35 | 55 | 0.87 |
| | Maltoma | 124 | 61 | 35 | 56 | |
| | Bone marrow | 74 | 39 | 24 | 49 | |
| | Other | 145 | 75 | 34 | 52 | |
| Presenting site | Nodal | 499 | 245 | 35 | 55 | 0.77 |
| | Extra-nodal | 343 | 175 | 33 | 53 | |
| Protocol | No | 292 | 195 | 10 | 38 | $0.2 \times 10^{-19}$ |
| | Yes | 538 | 214 | >60 | 64 | |
| SOC and grade | No, low grade | 118 | 62 | 27 | 50 | $<3.0 \times 10^{-20}$ |
| | No, high grade | 72 | 66 | 1 | 9 | |
| | Yes, low | 337 | 117 | >60 | 71 | |
| | Yes, high | 270 | 143 | 28 | 51 | |
| SOC and protocol | No, no protocol | 107 | 94 | 1 | 13 | $<3.0 \times 10^{-20}$ |
| | No, protocol | 94 | 42 | 35 | 59 | |
| | Yes, no protocol | 185 | 101 | 28 | 52 | |
| | Yes, protocol | 444 | 172 | >60 | 66 | |
| Protocol and grade | No, low grade | 136 | 72 | 28 | 52 | $<3.0 \times 10^{-20}$ |
| | No, high grade | 130 | 101 | 5 | 27 | |
| | Yes, low | 311 | 100 | >60 | 73 | |
| | Yes, high | 208 | 104 | 35 | 52 | |

SOC, specialist oncology centre.

an SOC and all but two received treatment as opposed to 13 of the 31 patients managed locally ($P<0.001$). Patients over 50 were less likely to be given chemotherapy ($P<0.001$). Eight of the 15 untreated patients were over 80 years of age. Patients managed at the SOC were significantly younger than those managed at 'other hospitals', 71% were aged less than 50 compared with 31% of patients managed at other hospitals.

*ML survival*

A total of 420 patients had died. Seventy-eight deaths were due to causes other than lymphoma. Seven patients died from intercurrent causes with no disease present, 40 patients died from intercurrent causes with active disease, 16 patients died from second malignancy and for 15 patients death was associated with the toxicity of therapy.

The 2 year survival, for the 842 patients, was 54% with a median survival time of 34 months. Survival curves for the prognostic factors are summarised in Table IV.

Patients treated at an SOC had a much improved prognosis compared to patients treated at 'other hospitals'. Patients treated according to a standard protocol had a better prognosis. The survival curves for each combination of the variables SOC, protocol and grade are shown in Figures 1 and 2.

As the survival curves by age revealed that there was little difference in the survival patterns for patients under 70 years, age was handled in the Cox analysis as a three category variable (<70, 70–79, >80). Stages 2, 3 and 5 had very similar survival patterns and hence stage was regrouped into five categories. As there was a very clear trend in survival, stage was handled as a continuous variable. Table V lists the most significant effect at each step of the Cox analysis. PS was the most significant variable related to survival. Several interaction effects were incorporated into the Cox model. These suggested that PS1 and PS2 patients had a better survival if treated by a recognised protocol but that protocol had little influence on the survival of patients with a low PS; the importance of protocol was most marked for patients treated at hospitals other than the SOC; the improved survival for patients treated at the SOC was particularly marked for high-grade patients. Stage, gender and age were also independently related to survival with survival being better for stage 1 patients and for females. Patients aged under 70 years fared better than patients aged 70–79 and those aged 80 and over.

*Leukaemia survival*

Thirty-two patients died from malignancy other than leukaemia and no patient died of a cause other than malignancy. Survival curves for the prognostic factors are summarised in Tables VI and VII.

*CLL* The overall median survival was 43 months, with a 2 year probability of survival of 61%. Social class 1 and 2 patients appeared to have a survival advantage compared to social class 3 to 5 patients ($P=0.018$). The survival curves for the combination of the variables SOC and protocol are shown in Figure 3. There were only ten patients who were treated at the SOC without a recognised protocol.

*ALL* The survival experience of 72 patients was analysed. The overall 2 year survival was 34% with a median survival

**Figure 1** Survival of low-grade lymphoma by specialist oncology centre (SOC) and protocol. Group 1, SOC and protocol; group 2, SOC and no protocol; group 3, protocol not SOC; group 4, not SOC and no protocol.

**Figure 2** Survival of high-grade lymphoma by specialist oncology centre (SOC) and protocol. Group 1, SOC and protocol; group 2, SOC and no protocol; group 3, protocol not SOC; group 4, not SOC and no protocol.

**Table V** Final Cox's model for survival for lymphoma patients

| *Variables* | P-*value* | *MLE of regression coefficient* | *s.e. of the estimate* |
|---|---|---|---|
| PS | $0.9\times10^{-18}$ | 0.562 | 0.2659 |
| Protocol and | $0.8\times10^{-14}$ | −1.3705 | 0.6073 |
| PS protocol[a] | | −0.0701 | 0.1654 |
| SOC and | $0.7\times10^{-16}$ | −1.0971 | 0.6725 |
| protocol SOC[a] | | 0.5654 | 0.2945 |
| Grade and | $0.6\times10^{-10}$ | 1.2380 | 0.4916 |
| SOC grade[a] | | −0.2698 | 0.2769 |
| Stage | 0.00012 | 0.2717 | 0.0749 |
| Gender | 0.0026 | −0.4784 | 0.1265 |
| Age <70 years | 0.0017 | −0.2630 | 0.2256 |
| 70–79 years | | 0.2904 | 0.2145 |

MLE, maximum likelihood estimate; s.e., standard error; PS, performance status; SOC, specialist oncology centre. [a]Denotes first-order interaction effect.

**Table VI** Variables examined for possible effect on survival from diagnosis for chronic lymphocytic leukaemia patients

| Variable | Level | Number of patients | Number of deaths | Median months | 2 year survival (%) | P-value |
|---|---|---|---|---|---|---|
| Age in years | <60 | 80 | 24 | 57 | 82 | 0.0001 |
| | 60-69 | 136 | 50 | 53 | 74 | |
| | 70-79 | 197 | 98 | 42 | 62 | |
| | ⩾80 | 113 | 78 | 13 | 37 | |
| Gender | Male | 295 | 148 | 40 | 61 | 0.25 |
| | Female | 231 | 102 | 48 | 65 | |
| PS | ⩾90% | 223 | 61 | >60 | 81 | 0.0001 |
| | 70-89% | 85 | 53 | 18 | 44 | |
| | <70% | 63 | 49 | 5 | 29 | |
| Social class | 1 and 2 | 85 | 37 | 50 | 72 | 0.12 |
| | 3 | 180 | 100 | 26 | 51 | |
| | 4 | 86 | 50 | 27 | 55 | |
| | 5 | 50 | 29 | 25 | 51 | |
| Protocol | Yes | 343 | 95 | 54 | 69 | 0.0001 |
| | No | 158 | 133 | 18 | 48 | |
| SOC | Yes | 60 | 28 | 57 | 68 | 0.20 |
| | No | 466 | 222 | 42 | 60 | |
| Protocol and SOC | Yes Yes | 50 | 22 | 57 | 72 | 0.0001 |
| | Yes No | 293 | 111 | 53 | 69 | |
| | No No | 148 | 89 | 18 | 47 | |

PS, performance status; SOC, specialist oncology centre.

**Table VII** Variables examined for possible effect on survival from diagnosis for acute lymphoblastic leukaemia patients

| Variable | Level | Number of patients | Number of deaths | Median months | 2 year survival (%) | P-value |
|---|---|---|---|---|---|---|
| Age in years | <25 | 23 | 12 | 29 | 56 | 0.0001 |
| | 25-54 | 16 | 9 | 29 | 55 | |
| | 55-74 | 19 | 19 | 2 | 5 | |
| | ⩾75 | 14 | 13 | 1 | 14 | |
| Gender | Male | 42 | 32 | 7 | 28 | 0.29 |
| | Female | 30 | 21 | 11 | 43 | |
| Social class | 1 and 2 | 12 | 10 | 3 | 33 | 0.88 |
| | 3 | 27 | 21 | 6 | 33 | |
| | 4 and 5 | 20 | 15 | 10 | 25 | |
| SOC | Yes | 41 | 25 | 22 | 45 | 0.0033 |
| | No | 31 | 28 | 3 | 19 | |
| Protocol | Yes | 55 | 37 | 15 | 43 | 0.0009 |
| | No | 14 | 13 | <1 | 7 | |
| Protocol and SOC | Yes/Yes | 27 | 16 | 23 | 46 | 0.013 |
| | Yes/No | 28 | 21 | 14 | 39 | |
| | No/No | 11 | 10 | 2 | 9 | |

SOC, specialist oncology centre.

time of 7 months. The survival curves for place of treatment are shown in Figure 4. Women appeared to have a survival advantage with 43% alive at 2 years *vs* 28% of men.

Fifty-four patients were coded as PS1. PS appeared to have a significant effect on survival with a median survival of 24 months for the PS1 patients *vs* 2 months for the 18 PS2 and PS3 patients.

There was a strong interaction between age at diagnosis and whether a patient was treated according to protocol. The majority of younger patients were treated according to protocol and fared best and those over 65 years of age and not so treated fared worst.

As there was little difference in the survival patterns of leukaemia patients aged under 80 years, age was handled in the Cox analysis as a two category variable (<80, >80). Table VIII lists the most significant effect at each step of the Cox multivariate analysis. PS was the most significant variable related to survival, followed by grade. Several interaction effects were incorporated into the model. These suggested that PS2 and PS3 patients had a better survival if treated by a recognised protocol, patients aged 80 and over who were not treated according to a recognised protocol had a very poor prognosis, and an improved prognosis for females was most marked for PS3 patients.

### Discussion

### *Data*

A specialist database was set up for the study of haematological malignancies to make ascertainment more timely and to ensure diagnostic accuracy. It was also felt that ascertainment by the NWRCR of low-grade conditions in the elderly, which may not have required hospital admission, may have been incomplete.

**Figure 3** Survival of chronic lymphocytic leukaemia by specialist oncology centre (SOC) and protocol. Group 1, SOC and protocol; group 2, protocol not SOC; group 3, not SOC and no protocol.

**Figure 4** Survival of acute lymphoblastic leukaemia by specialist oncology centre (SOC). Group 1, SOC; group 2, not SOC.

Estimates of completeness have been made for the NWRCR (Benn *et al.*, 1982; Nwene and Smith, 1982) and for the national cancer registration system (Swerdlow *et al.*, 1993) and both found ascertainment levels for lymphomas of about 95%. Cross-validation checks with both the NWRCR and some laboratories showed that additional cases were included in the study and accuracy improved. However some cases of CLL diagnosed on a blood count had not been notified. The level of ascertainment of cases of ML and ALL, therefore is high and the study represents one of the few to attempt to capture a high proportion of cases of CLL.

The diagnosis of all cases was validated and a high proportion of diagnostic material was reviewed. The decision to

**Table VIII** Final Cox's model for survival for leukaemia patients

| *Variables* | P-*value* | *MLE of regression coefficient* | *s.e. of the estimate* |
|---|---|---|---|
| PS | $0.5 \times 10^{-26}$ | 1.0777 | 0.4043 |
| Grade | $0.7 \times 10^{-6}$ | 0.9298 | 0.1885 |
| Protocol and | 0.00015 | 1.0921 | 0.5480 |
| PS protocol[a] | | −0.1244 | 0.1897 |
| Age and | 0.000088 | 1.6967 | 0.5562 |
| protocol age[a] | | −0.8407 | 0.3550 |
| Gender and | 0.0029 | −0.2766 | 0.3580 |
| PS gender[a] | | −0.0528 | 0.1704 |

MLE, maximum likelihood estimate; s.e., standard error; PS, performance status. [a]Denotes first-order interaction effect.

limit the survival analysis of ML to those where pathology review had been carried out restricted the data set, particularly with regard to patients diagnosed during 1983 and 1984. All the factors where cases without pathology review differed from those with review, that is older age, a lower probability of being treated according to a recognised protocol and a lower probability of referral to the SOC, would be likely to bias the results towards a better outcome. Differences in survival shown by this study are therefore likely to have been decreased by restricting the data set.

### *Diagnosis*

Dick *et al.* (1987*b*) reported 'unconventional' or admixed diagnostic terminology that did not conform to any of the classification schemes currently in use. This problem led to errors of coding to ICDO. The same problem contributed to our decision to exclude cases without review. The findings from the above study suggested that the unreliability of subtyping of ML was at least 40% although concurrence could be as high as 80% for some categories. In another series complete concordance of diagnosis was achieved between submitting pathologists and a lymphoma panel in just over half the cases (Bird *et al.*, 1984). The results of these studies are similar to our findings of 47% reclassification and 25% major revision.

In a two level pathology review, good concordance was found between the regional centre pathologist and the panel and for the most common lymphomas a second review by panel was considered unnecessary (Wolf *et al.*, 1988). Kim *et al.* (1982) found a discordance of 9% between the regional centre pathologists and a panel. ML cases in our study were reviewed by two regional centre pathologists.

Nearly 25% of the changes in diagnosis on review had clinical relevance. Without such histology review many ML patients may not receive the most appropriate therapy.

Bartl *et al.* (1988) found that 12% of trephines were unclassifiable and a concordance of 76% was found when lymph node material was available for comparison. Many of the ML patients in our study diagnosed on bone marrow examination only had other tissue available for biopsy. Chemotherapy regimens given to these patients may not have been the most appropriate.

### *Staging*

The problems surrounding the reconstruction of clinical stage of disease resulted in a two tier staging system. Analysis of treatment was carried out on the basis that the information available in the case notes was the information available to the clinician when deciding on a treatment plan. However it is possible that further information was available to the clinician that was not recorded. No account could be taken of silent case records in the analysis. The method of defining stage used in the study did appear to enable stratification by stage in the analysis in an appropriate manner.

The problem of silent records is a major one for studies of health care. Feigl *et al.* (1988) found that clinical stage was not routinely documented but that extent of disease could be coded in most cases on a crude staging system. Data related to clinician observation, especially performance status was frequently not available. These findings are similar to the findings of our study.

Data may be missing from the hospital records for two reasons, either the procedure or observation that should have occurred did not do so, or the result was not recorded. Laboratory and other diagnostic procedures are generally reported in the case notes and if the results of a specific test are not recorded then it is reasonable to assume that the test was not done. Over a quarter of ML cases were insufficiently investigated to allow full clinical staging before treatment, which for many of them was intensive therapy. The survival of cases designated as stage 5 was similar to that of Ann Arbor stage 2 and 3 patients suggesting that this group included good prognosis patients who had not been fully evaluated.

*Survival*

Published studies give 5 year survival rates for high and intermediate grade patients of 50% (Cowan *et al.*, 1989). An EORTC trial showed a 35% 5 year survival for high-grade patients with 5 year survival for low-grade patients of between 50% and 80%, depending on whether the cell pattern was follicular or diffuse (Somers *et al.*, 1987). The 2-year survival rate for high-grade patients in our study was 42% and 66% for low-grade. The median survival for one series of patients with ALL was 18 months (Barnett *et al.*, 1986) and 23 months in another series (Marcus *et al.*, 1986) compared with a median survival for our study of 7 months for all ALL patients and 22 months for ALL patients managed at the SOC.

*Age*

Elderly patients are rarely included in clinical trials and such patients may be undertreated or inappropriately treated (Tirelli *et al.*, 1988; Fentiman *et al.*, 1990; Fentiman, 1991). A recent EORTC consensus meeting on neoplasia in the elderly (Monfardini and Chabner, 1991) concluded that elderly patients should receive maximal curative treatment on accepted protocols and that adjustments to protocols should not be based on age alone. ML patients presenting over the age of 65 with high-grade disease and treated with protocols specifically designed for these patients are reported as achieving 5 year survival rates of 38–44% (Vose *et al.*, 1989; O'Reilly and Connors, 1992*b*). These survival rates appear to exceed the survival of all high grade patients in this study, however such reports confirm our findings that elderly patients can do well.

Tirelli *et al.* (1988) found that 44% of patients in an EORTC study of patients aged 70 or over were treated conservatively and 56% were treated aggressively. The corresponding proportions for our study were 39% and 25%. The overall median survival was 37 months, again markedly in excess of our study. Patients with ALL over the age of 55 were less likely to be referred to the SOC and less likely to be given chemotherapy at other hospitals. Taylor *et al.* (1992) in a population-based study of patients aged 60 and over with ALL concluded that the prognosis was poor but that age itself should not be a bar to therapy as a small proportion can have a good survival. These findings are similar to our own where a few elderly patients had long-term survival. It seems likely that many elderly patients are being undertreated.

*Place of treatment*

The proportion of patients recorded as entered into randomised clinical trials was low. Patients were unlikely to have been entered into a trial unless they had been treated at the SOC. There did appear to be a possible survival advantage for clinical trial entry. A higher level of protocol violations would be probable if trial entry is not recorded.

The debate about whether clinical trial entry improves survival has continued for many years and the available data are still inadequate. Stiller (1992) extensively reviewed the available literature in relation to treatment at specialist centres or within clinical trials. This review concluded that for the majority of cancers, the greater clinical experience and standardisation of treatment, whether in the context of clinical trials or specialist treatment centres or both, is of direct benefit to patients.

Karjalainen and Palva (1989) found that patients with multiple myeloma had an improved survival if they lived in an area where clinical trial entry was the treatment policy. The authors concluded that there was a greater level of uniformity in the trial areas where treatment was according to a recognised protocol and this was the key factor in an improved survival. In our study the major benefit to patients was treatment according to an appropriate standard protocol but for some ML patients and ALL patients there was an additional advantage from treatment at a SOC. The importance of the use of an appropriate protocol was marked for patients managed at 'other hospitals'. CLL is frequently perceived as a low-grade disease of the elderly and patients are rarely managed at a SOC but patients not treated according to a protocol fared badly.

Rosenberg (1986), in a review of therapy for Hodgkin's disease, noted that increasing numbers of patients were being treated in small community hospitals and felt that this trend would be detrimental to patient management. Also fewer patients would be entered into clinical trials and therefore it would be harder to improve survival and decrease therapy-related morbidity. The improvement of results with experience provides one of the strongest justifications for encouraging referral of patients to centres with a specialist interest in their management (Oliver, 1986). The majority of hospital groups in this study were responsible for the management of fewer than five patients a year and very few such patients were entered into clinical trials. The survival advantage from management at the SOC for ALL and some ML patients demonstrates the benefits of experience and specifically that staging is likely to be more precise and treatment protocols more appropriate for specific patients. Complications of therapy may also be recognised earlier. In addition the greater experience may allow the use of less therapy without compromising survival, reducing the frequency of the late effects of treatment. There may also be improved follow-up and detection of late effects.

*Conclusion*

Histology review and staging are necessary if patients are to be appropriately treated. Survival is improved if treatment is given according to a current standard protocol and may be further improved by treatment at a SOC. Specific studies need to be defined for determining the best treatment for elderly patients. Although this study has its limitations it is an additional contribution to the few population-based studies of adult cancer.

**Acknowledgements**

We are grateful for the full cooperation of all the histopathologists and haematologists who participated in this study and to all the clinicians who allowed their patients to be entered into the study. We are also grateful for the continued help and support of the Manchester Lymphoma Group. We also thank Mrs Jennie Spencer for data collection and data entry, all the medical records staff throughout the North West Region and the staff of the North West Regional Cancer Registry. The study was supported by a grant from the North West Regional Research and Development Fund.

**References**

BARTL R. HANSMANN M-L. FRISCH B AND BURKHARDT R. (1988). Comparative histology of malignant lymphomas in lymph node and bone marrow. *Br. J. Haematol.*, **69**, 229-237.

BARNETT MJ. GREAVES MF. AMESS JA. GREGORY WM. ROHATINER AZ. DHALIWAL HS. SLEVIN ML. BIRULS R. MALPAS JS AND LISTER TA. (1986). Treatment of acute lymphoblastic leukaemia in adults. *Br. J. Haematol.*, **64**, 455-468.

BENN RT. LECK I AND NWENE UP. (1982). Estimation of completeness of cancer registration. *Int. J. Epidemiol.*, **11**, 362-367.

BERKSON J AND GAGE RP. (1950). Calculation of survival rates for cancer. *Proceedings of Staff Meetings. Mayo Clinic*, **25**, 270-286.

BIRD CC. LAUDER I. KELLETT HS. CHORLTON I. BARNES N. DARWIN C. CARTWRIGHT RA AND BOYKO R. (1984). Yorkshire Regional lymphoma histology panel. Analysis of five years experience. *J. Pathol.*, **143**, 249-258.

BMDP. (1992). *Biomedical Computer Programs P-Series*. University of California Press: Berkeley, USA.

CARBONE PP. KAPLAN HS. MUSSHOFF K. SMITHERS DW AND TUBIANA M. (1971). Report of the committee on Hodgkin's disease staging classification. *Cancer Res.*, **31**, 1860-1861.

COWAN RA. JONES M. HARRIS M. STEWARD WP. RADFORD JA. WAGSTAFF J. DEAKIN DP AND CROWTHER D. (1989). Prognostic factors in high and intermediate grade non-Hodgkin's lymphoma. *Br. J. Cancer*, **59**, 276-282.

COX DR. (1972). Regression models and life-tables. *J. R. Stat. Soc., Series B*, **34**, 187-200.

DICK FR. VAN LIER S. BANKS P. FRIZZERA G. WITRAK G. GIBSON R. EVERETT G. SCHUMAN L. ISACSON P. O'CONOR G. CANTOR K. BLATTNER W AND BLAIR A. (1987*a*). Use of the Working Formulation for non-Hodgkin's lymphoma in epidemiologic studies: agreement between reported diagnoses and a panel of experienced pathologists. *J. Natl Cancer Inst.*, **78**, 1137-1146.

DICK FR. VAN LIER S. MCKEEN K. EVERETT G AND BLAIR A. (1987*b*). Nonconcurrence in abstracted diagnoses of non-Hodgkin's lymphoma. *J. Natl Cancer Inst.*, **78**, 675-677.

ERSBOLL J. SCHULTZ HB. HOUGAARD P. NISSEN NI AND HOU-JENSEN K. (1985). Comparison of the Working Formulation of non-Hodgkin's lymphoma with the Rappaport, Kiel and Lukes and Collins classifications. Translational value and prognostic significance based on review of 658 patients treated at a single institution. *Cancer*, **55**, 2442-2458.

FEIGL P. GLAEFKE G. FORD L. DIEHR P AND CHU J. (1988). Studying patterns of cancer care: how useful is the medical record? *Am. J. Public Health*, **78**, 526-533.

FENTIMAN IS. (1991). Treatment of cancer in the elderly. Guest editorial. *Br. J. Cancer*, **64**, 993-995.

FENTIMAN IS. TIRELLI U. MONFARDINI S. SCHNEIDER M. FESTEN J. COGNETTI F AND AAPRO MS. (1990). Cancer in the elderly. Why so badly treated? *Lancet*, **335**, 1020-1022.

GERARD-MARCHANT R. HAMLIN I. LENNERT K. RIELKE F. STANSFELD AG AND VAN UNNIK JAM. (1974). Classification of non-Hodgkin's lymphoma (letter). *Lancet*, **2**, 406-408.

KARJALAINEN S AND PALVA I. (1989). Do treatment protocols improve end results? A study of survival of patients with multiple myeloma in Finland. *Br. Med. J.*, **299**, 1069-1072.

KIM H. ZELMAN RJ. FOX MA. BARNETT JM. BERARD CW. BUTLER JJ. BYRNE Jr GE. DORFMAN RF. HARTSTOCK RJ. MANN RB. NEIMAN RS. REBUCK JW. SHEEHAN WW. VARIAKOJIS D. WILSON JF AND RAPPAPORT H. (1982). Pathology panel for lymphoma clinical stuies. A comprehensive analysis of cases accumulated since its inception. *J. Natl Cancer Inst.*, **68**, 43-67.

MARCUS RE. CATOVSKY D. JOHNSON SA. GREGORY WM. TALAVERA JG. GOLDMAN JM AND GALTON DA. (1986). Adult acute lymphoblastic leukaemia: a study of prognostic features and response to treatment over a ten year period. *Br. J. Cancer*, **53**, 175-180.

MCILLMURRAY MB. (1987). District cancer physicians. Report of a working group of the Association of Cancer Physicians. *J. R. Coll. Physicians London*, **21**, 117-121.

MEAD GM. (1987). Malignant lymphoma: a clinicians view. *J. Pathol.*, **151**, 179-182.

MONFARDINI S AND CHABNER B. (1991). Joint NCI-EORTC consensus meeting on neoplasia in the elderly. *Eur. J. Cancer*, **27**, 653-654.

NWENE UP AND SMITH A. (1982). Assessing completeness of cancer registration in the North Western Region of England by a method of independent comparison. *Br. J. Cancer*, **46**, 635-639.

O'REILLY SE AND CONNORS JM. (1992*a*). Non-Hodgkin's lymphoma. I. Characterisation and treatment. Currrent issues in cancer. No. 8. *Br. Med. J.*, **304**, 1682-1686.

O'REILLY SE AND CONNORS JM. (1992*b*). Non-Hodgkin's lymphoma. II. Management problems. Current issues in cancer. No. 9. *Br. Med. J.*, **305**, 39-42.

OLIVER RTD. (1986). Rare cancers and specialist centres. *Br. Med. J.*, **292**, 641-642.

PETO R AND PETO J. (1972). Asymptotically efficient rank variant test procedures. *J. R. Stat. Soc., Series A.*, **135**, 185-206.

ROSENBERG SA. (1986). Hodgkin's disease: no stage beyond cure. *Hosp. Pract.*, **21**, 91-108.

SIMON R. DURRLEMAN S. HOPPE RT. BONADONNA G. BLOOMFIELD C. RUDDERS RA. CHESON BD AND BERARD CW. (1988). The non-Hodgkin's lymphoma pathologic classification project. Long term follow-up of 1153 patients with non-Hodgkin's lymphomas. *Ann. Int. Med.*, **109**, 939-945.

SOMERS R. BURGERS JMV. QASIM M. VAN GLABBEKE M. DUEZ N AND HAYAT M. (1987). EORTC trial non-Hodgkin lymphomas. *Eur. J. Cancer Clin. Oncol.*, **23**, 283-293.

STILLER CA. (1989). Survival of patients with cancer. *Br. Med. J.*, **299**, 1058-1059.

STILLER CA. (1992). Survival of patients in clinical trials and at specialist centres. In *Introducing New Treatments for Cancer. Practical, Ethical and Legal Problems*. Williams CJ (ed.) pp. 119-136. John Wiley: Chichester, UK.

SWERDLOW AJ. DOUGLAS AJ. VAUGHAN HUDSON G AND VAUGHAN HUDSON B. (1993). Completeness of cancer registration in England and Wales: an assessment based on 2145 patients with Hodgkin's disease independently registered by the British National Lymphoma Investigation. *Br. J. Cancer*, **67**, 326-329.

TAYLOR PRA. REID MM. BROWN N. HAMILTON PJ AND PROCTOR SJ. (1992). Acute lymphoblastic leukaemia in patients aged 60 years and over: a population based study of incidence and outcome. *Blood*, **80**, 1813-1817.

TIRELLI U. ZAGONEL V. SERRAINO J. THOMAS J. HOERNI B. TANGURY A. RUHL U. BEY P. TUBIANA N. BREED WPM. ROOZENDAAL KJ. HAGENBEEK A. HUPPERETS PS AND SOMERS R. (1988). Non-Hodgkin's lymphomas in 137 patients aged 70 years or older: a retrospective European Organisation for Research and Treatment of Cancer Lymphoma Group study. *J. Clin. Oncol.*, **6**, 1708-1713.

VOSE JM. ARMITAGE JO. WEISENBURGER DD. BIERMAN PJ. SORENSON S AND HUTCHINS M. (1989). The importance of age in survival of patients treated with aggressive non-Hodgkin's. *J. Clin. Oncol.*, **6**, 1838-1844.

WANG Y. (1986). Classification of non-Hodgkin's lymphoma. *Am. J. Roentgenol.*, **147**, 205-208.

WOLF BC. GILCHRIST KW. MANN RB AND NIEMAN RS. (1988). Evaluation of pathology review of malignant lymphomas and Hodgkin's disease in cooperative clinical trials. The Eastern Cooperative Oncology Group experience. *Cancer*, **62**, 1301-1305.

WORLD HEALTH ORGANIZATION. (1976). *International Classification of Diseases for Oncology*. World Health Organization: Geneva.

YOUNGSON JHAM. (1984). The epidemiology of lymphoid malignancy in the north-west of England (Ph.D. thesis). Faculty of Medicine, University of Manchester: Manchester.